# Retraction: Ivermectin induces autophagy-mediated cell death through the AKT/mTOR signaling pathway in glioma cells

**DOI:** 10.1042/BSR20192489_RET

**Published:** 2026-03-26

**Authors:** Aili Gao, Jingjing Liu, Hongsheng Liang, Chen Chen, Xiaoxing Wang, Faling Qu, Haiyang Wang, Kongbin Yang, Qing Wang, Ning Zhao, Jing Meng

**Affiliations:** School of Life Science, Northeast Agricultural University, Harbin, Heilongjiang, China

**Keywords:** AKT/mTOR, apoptosis, autophagy, Glioma, Ivermectin

This article is being retracted from *Bioscience Reports* at the request of the Editor-in-Chief and the Editorial Board. This follows the receipt of a notification from a reader, alerting the Editorial Board to similarities between this article and one published in the *Journal of Cancer* (2020) by overlapping authors (DOI: 10.7150/jca.46697). During the investigation, the Editorial Office and the authors identified additional similarities.

Specifically:

- The Figure 1A control panels appear in both articles
- A degree of similarity has been noted between the Figure 3A AKT and mTOR bands of this article (after a mirror-image rotation of the mTOR bands).
- The Figure 5C Control and CQ scatter plot panels also appear in Figure 4C of DOI: 10.7150/jca.46697.
- The Figure 5C IVM + CQ/U251and IVM + CQ/C6 scatterplot panels also appear as the Figure 4C MOX/U251 and MOX + CQ/U251 panels respectively in DOI: 10.7150/jca.46697.
- Regions of the Figure 7D Ki67/Control panel overlap with regions of the Figure 6D Ki67/Control and CQ panels of DOI: 10.7150/jca.46697

The authors cooperated with the investigation. They provided raw data for many of the figures and disagree with the similarities noted between the Figure 3A western blot bands (for which raw blots were provided for all except the mTOR bands). They explained that the image similarities were due to oversights during export and figure preparation of the two manuscripts. They also noted an erratum that has been published in the *Journal of Cancer* that replaces various figure panels of Figure 4C. However, whilst investigating the new data, new concerns have arisen, and it was not felt that the erratum alleviated the original concerns.

Given the lack of raw data for the mTOR bands and concerns over original and new data, the Editorial Board has lost confidence in the validity of the data and stands by the decision to retract. All authors were contacted regarding the retraction, with one agreeing to the action being taken. All other authors either disagree or did not comment.

